# Dichlorido{[(diphenyl­phosphino)meth­yl]bis­(2-methyl­phen­yl)phosphine-κ^2^ *P*,*P*′}palladium(II)

**DOI:** 10.1107/S1600536809036678

**Published:** 2009-09-19

**Authors:** Ansonia H. Badgett, Danielle L. Gray, Quinetta D. Shelby

**Affiliations:** a DePaul University, Department of Chemistry, 1110 West Belden Avenue, Chicago, Illinois 60614, USA; b University of Illinois, School of Chemical Sciences, Box 59-1, 505 South Mathews Avenue, Urbana, Illinois 61801, USA

## Abstract

In the title compound, [PdCl_2_(C_27_H_26_P_2_)] or PdCl_2_[(C_6_H_5_)_2_PCH_2_P(C_6_H_4_CH_3_)_2_], the palladium center has a distorted square-planar geometry. There are two crystallographically independent mol­ecules in the asymmetric unit. The dihedral angle between the PdP_2_ and PdCl_2_ planes is 2.95 (4)° in one independent mol­ecule and 5.15 (4)° in the other. The P—Pd—P and P—C—P bond angles are significantly distorted because of the small bite angle of the chelating (bis­phosphino)methane ligand. The steric demands of the substituted rings in the mixed ligand cause a slight elongation of the Pd—P(C_6_H_4_CH_3_)_2_ bond relative to the Pd—P(C_6_H_5_)_2_ bond. In one molecule the tolyl ring shows positional disorder in a 0.58 (2):0.42 (2) ratio, in the other molecule the phenyl ring shows positional disorder in a 0.838 (9):0.162 (9) ratio.

## Related literature

For the steric effects of (bis­phosphino)methane ligands, see: Filby *et al.* (2006 ▶); Dossett *et al.* (2001 ▶). For dichlorido[bis­(diphenyl­phosphino)methane]palladium(II), see: Shahid *et al.* (2009 ▶); Steffen & Palenik (1976 ▶). For dichlorido[bis­(dicyclo­hexyl­phosphino)methane]palladium(II), see: Mague *et al.* (2007 ▶). For related literature regarding the synthesis of the title compound, see: Wass (2001 ▶); Gauthron *et al.* (1998 ▶).
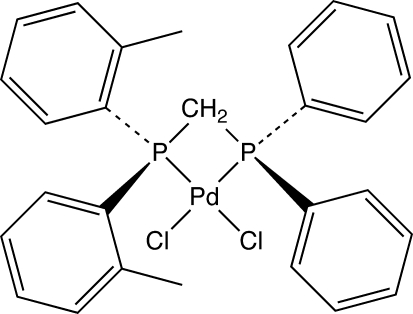


## Experimental

### 

#### Crystal data

[PdCl_2_(C_27_H_26_P_2_)]
*M* *_r_* = 589.72
Monoclinic, 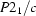

*a* = 17.8761 (10) Å
*b* = 16.7568 (9) Å
*c* = 16.9407 (9) Å
β = 90.446 (3)°
*V* = 5074.4 (5) Å^3^
*Z* = 8
Mo *K*α radiation
μ = 1.08 mm^−1^
*T* = 193 K
0.30 × 0.28 × 0.26 mm

#### Data collection

Bruker Kappa APEXII CCD diffractometer
Absorption correction: integration (*SHELXTL*/*XPREP*; Bruker, 2005 ▶ and *SADABS*; Bruker, 2007 ▶)*T*_min_ = 0.728, *T*_max_ = 0.801
83307 measured reflections
9350 independent reflections
8400 reflections with *I* > 2σ(*I*)
*R*_int_ = 0.037

#### Refinement

*R*[*F* ^2^ > 2σ(*F* ^2^)] = 0.030
*wR*(*F* ^2^) = 0.074
*S* = 1.26
9350 reflections
702 parameters
469 restraints
H-atom parameters constrained
Δρ_max_ = 0.66 e Å^−3^
Δρ_min_ = −0.47 e Å^−3^

### 

Data collection: *APEX2* (Bruker, 2004 ▶); cell refinement: *SAINT* (Bruker, 2005 ▶); data reduction: *SAINT* and *XPREP*/*SHELXTL* (Sheldrick, 2008 ▶); program(s) used to solve structure: *SHELXTL*; program(s) used to refine structure: *SHELXTL*; molecular graphics: *SHELXTL* and *CrystalMaker* (*CrystalMaker*, 1994 ▶); software used to prepare material for publication: *XCIF*/*SHELXTL*.

## Supplementary Material

Crystal structure: contains datablocks I, global. DOI: 10.1107/S1600536809036678/ez2183sup1.cif

Structure factors: contains datablocks I. DOI: 10.1107/S1600536809036678/ez2183Isup2.hkl

Additional supplementary materials:  crystallographic information; 3D view; checkCIF report

## Figures and Tables

**Table 1:** Hydrogen-bond geometry (Å, °)

*D*—H⋯*A*	*D*—H	H⋯*A*	*D*⋯*A*	*D*—H⋯*A*
C14—H14*A*⋯Cl1	0.98	2.63	3.612 (4)	179
C34—H34*A*⋯Cl3	0.98	2.66	3.637 (4)	179

**Fig. 1.**
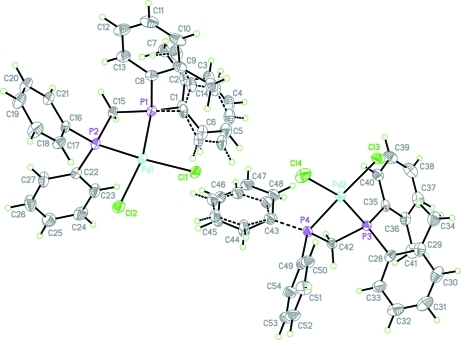
Two independent molecular structures of the title compound in the asymmetric unit showing disorder of the ortho-tolyl ring C1 to C7 in molecule 1 (left) and the phenyl ring C43 to C48 in molecule 2 (right) with 35% probability ellipsoids for non-H atoms and circles of arbitrary size for H atoms.

**Fig. 2.**
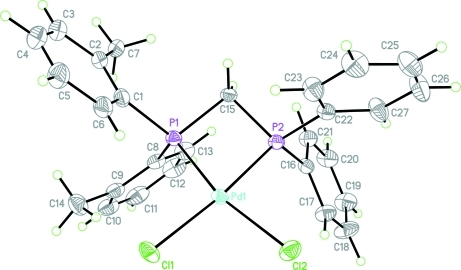
Molecule 1 of the title compound showing 35% probability ellipsoids for non-H atoms and circles of arbitrary size for H atoms. Disorder of the ortho-tolyl ring C1 to C7 has been omitted for clarity.

**Fig. 3.**
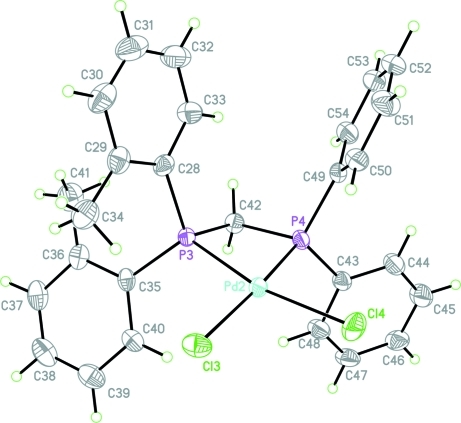
Molecule 2 of the title compound showing 35% probability ellipsoids for non-H atoms and circles of arbitrary size for H atoms. Disorder of the phenyl ring C43 to C48 has been omitted for clarity.

## supplementary crystallographic information

### Comment

The steric effects on the bite angles and preferred bonding modes of aromatic (bisphosphino)methane ligands have been studied (Filby *et al.*, 2006), and there is interest in their four-membered palladacyclic complexes because they have been shown to be active catalysts for reactions such as the copolymerization of CO and C_2_H_4_ (Dossett *et al.*, 2001).

The title compound, PdCl_2_[(C_6_H_5_)_2_PCH_2_P(C_6_H_4_CH_3_)_2_], crystallizes with two independent molecules in the asymmetric unit with the palladium center in each adopting a distorted square-planar geometry. The C1 to C7 *ortho*-tolyl ring in molecule 1 and the C43 to C48 phenyl ring in molecule 2 are disordered. The asymmetric unit containing both molecules showing disorder of their respective aromatic rings is shown in Fig. 1. In molecule 1 (Fig. 2) the dihedral angle between the PdP_2_ and PdCl_2_ planes is 2.95 (4)°, whereas the dihedral angle is 5.15 (4)° in molecule 2 (Fig. 3). The related symmetrical compounds PdCl_2_[(C_6_H_5_)_2_PCH_2_P(C_6_H_5_)_2_] (Shahid *et al.*, 2009; Steffen & Palenik, 1976) and PdCl_2_[(C_6_H_11_)_2_PCH_2_P(C_6_H_11_)_2_] (Mague *et al.*, 2007) show similar deviations from planarity about the palladium center due partly to the small bite angle of the chelating (bisphosphino)methane ligand and the spatial demands of the ligand substituents. The P—Pd—P bond angles of 73.75 (3)° in molecule 1 and 73.37 (3)° in molecule 2 are significantly distorted from the normal square-planar value of 90°, and the P—C—P bond angles (93.95 (13) and 92.73 (14)°, respectively) show major deviations from the normal tetrahedral value of 109.5°. Notably, the Pd—P(C_6_H_4_CH_3_)_2_ bond lengths of 2.2646 (8) in molecule 1 and 2.2604 (8) Å in molecule 2 are slightly longer than the Pd—P(C_6_H_5_)_2_ bond lengths (2.2197 (7) and 2.2146 (8) Å in the respective molecules), which is likely a result of the steric requirements of the substituted rings.

There are two short Cl···H interactions: Cl1···H14A—C14 is 2.631 Å and Cl3···H34A—C34 is 2.658 Å. These are intramolecular Cl···H—C hydrogen bonds and they occur between the same atoms on both symmetrically independent molecules. The Cl···H—C angles are nearly linear (178.9° and 178.7°, respectively).

### Experimental

The mixed ligand Ph_2_PCH_2_P(C_6_H_4_CH_3_)_2_ has been reported in the patent literature (Wass, 2001), and our synthetic procedure will be published elsewhere. The title compound, PdCl_2_[(C_6_H_5_)_2_PCH_2_P(C_6_H_4_CH_3_)_2_], was prepared from a procedure adapted from that described previously for the synthesis of PdCl_2_[(C_6_H_5_)_2_PCH_2_P(C_6_H_5_)_2_] by Gauthron *et al.* (1998). A solid mixture of Ph_2_PCH_2_P(C_6_H_4_CH_3_)_2_ (612 mg, 1.48 mmol) and PdCl_2_ (260 mg, 1.47 mmol) was suspended in 50% EtOH (25 ml) and concentrated HCl (25 ml). The mixture was refluxed overnight, and a yellow solid precipitated from solution. The mixture was filtered, and the yellow solid was washed with H_2_O (2 × 25 ml) and EtOH (2 × 25 ml). After drying under vacuum, the title compound was obtained as a yellow powder (0.844 g, 98%). Mp: 258.1 °C (dec.). Anal. Calcd for C_27_H_26_Cl_2_P_2_Pd: C, 54.99; H, 4.44; P, 10.50. Found: C, 54.84; H, 4.33; P, 10.18. ^1^H NMR (CDCl_3_): δ 2.27 (s, C*H*,_3_), 4.30 (virtual t, ^2^*J*_HP_ = 10.6, 9.0 Hz, C*H*_2_), 7.23–8.04 (m, C_6_*H*_5_ and C_6_*H*_4_CH_3_). ^31^P{^1^H} NMR (CDCl_3_): δ -54.1 (d, ^2^*J*_PP_ = 88 Hz, *P*(C_6_H_5_)_2_ or *P*(C_6_H_4_CH_3_)_2_), -52.9 (d, ^2^*J*_PP_ = 88 Hz, *P*(C_6_H_5_)_2_ or *P*(C_6_H_4_CH_3_)_2_).

Single crystals suitable for X-ray diffraction were grown from slow diffusion of pentane into a concentrated CH_2_Cl_2_ solution at room temperature.

### Refinement

The proposed structural model consisting of two independent host molecules that each exhibit disorder off of the phosphines was developed. The positional disorder present in molecule 1 (Fig. 1) is located on the *ortho*-tolyl ring that contains C1 to C7. The disordered *ortho*-tolyl rings were restrained to have similar P—C bond distances, the same geometries, and to be flat using an effective standard deviation (e.s.d.) for each restraint of 0.01 Å. The final refinement showed that the *ortho*-tolyl ring is located in the primary position 57.8 (20)% of the time. The disorder present in molecule 2 (Fig. 1) is located on the phenyl ring that contains C43 to C48. The disordered phenyl rings were restrained to have similar P—C bond distances, similar C—C bond distances across the ring, and to be flat using e.s.d.'s of 0.01. The final refinement showed only a slight disorder in the phenyl ring with the primary position being 83.83 (9)% occupied. Anti bumping restraints were also used to prevent close contacts between the H atoms on the P—C—P bridged carbon and the *ortho* H atoms on the phenyl rings. Rigid-bond restraints (e.s.d. 0.01) were imposed on displacement parameters for all disordered sites and similar displacement amplitudes (e.s.d. 0.01) were imposed on disordered sites overlapping by less than the sum of Van der Waals radii. Methyl H atom positions, R—CH_3_, were optimized by rotation about R—C bonds with idealized C—H, R—H and H···H distances (C—H = 0.9800 and H···H = 1.6000 Å). Remaining H atoms were included as riding idealized contributors (aromatic C-H = 0.9500 Å, and R_2_CH_2_ C-H = 0.9900 Å). Methyl H atom U's were assigned as 1.5 times *U*_eq_ of the carrier atom; remaining H atom U's were assigned as 1.2 times carrier *U*_eq_.

### Crystal data

**Table:**

[PdCl_2_(C_27_H_26_P_2_)]	*F*(000) = 2384
*M**_r_* = 589.72	*D*_x_ = 1.544 Mg m^−^^3^
Monoclinic, *P*2_1_/*c*	Mo *K*α radiation, λ = 0.71073 Å
Hall symbol: -P 2ybc	Cell parameters from 9741 reflections
*a* = 17.8761 (10) Å	θ = 2.4–27.1°
*b* = 16.7568 (9) Å	µ = 1.08 mm^−^^1^
*c* = 16.9407 (9) Å	*T* = 193 K
β = 90.446 (3)°	Prism, yellow
*V* = 5074.4 (5) Å^3^	0.30 × 0.28 × 0.26 mm
*Z* = 8	

### Data collection

**Table:**

Bruker Kappa APEXII CCD diffractometer	9350 independent reflections
Radiation source: fine-focus sealed tube	8400 reflections with *I* > 2σ(*I*)
graphite	*R*_int_ = 0.037
profile data from φ and ω scans	θ_max_ = 25.4°, θ_min_ = 1.7°
Absorption correction: integration (*SHELXTL*/*XPREP*; Bruker, 2005)	*h* = −20→21
*T*_min_ = 0.728, *T*_max_ = 0.801	*k* = −20→20
83307 measured reflections	*l* = −20→20

### Refinement

**Table:**

Refinement on *F*^2^	Primary atom site location: structure-invariant direct methods
Least-squares matrix: full	Secondary atom site location: difference Fourier map
*R*[*F*^2^ > 2σ(*F*^2^)] = 0.030	Hydrogen site location: inferred from neighbouring sites
*wR*(*F*^2^) = 0.074	H-atom parameters constrained
*S* = 1.26	*w* = 1/[σ^2^(*F*_o_^2^) + (0.0262*P*)^2^ + 5.*P*] where *P* = (*F*_o_^2^ + 2*F*_c_^2^)/3
9350 reflections	(Δ/σ)_max_ = 0.006
702 parameters	Δρ_max_ = 0.66 e Å^−^^3^
469 restraints	Δρ_min_ = −0.47 e Å^−^^3^

### Special details

**Table:**

Experimental. One distinct cell was identified using *APEX2* (Bruker, 2004). Six frame series were integrated and filtered for statistical outliers using *SAINT* (Bruker, 2005) then corrected for absorption by integration using *SHELXTL*/*XPREP* V2005/2 (Bruker, 2005) before using *SAINT*/*SADABS* (Bruker, 2007) to sort, merge, and scale the combined data. No decay correction was applied.
Geometry. All e.s.d.'s (except the e.s.d. in the dihedral angle between two l.s. planes) are estimated using the full covariance matrix. The cell e.s.d.'s are taken into account individually in the estimation of e.s.d.'s in distances, angles and torsion angles; correlations between e.s.d.'s in cell parameters are only used when they are defined by crystal symmetry. An approximate (isotropic) treatment of cell e.s.d.'s is used for estimating e.s.d.'s involving l.s. planes.
Refinement. Structure was phased by direct methods (Sheldrick, 2008). Systematic conditions suggested the unambiguous space group. The space group choice was confirmed by successful convergence of the full-matrix least-squares refinement on *F*^2^. The highest peaks in the final difference Fourier map were in the vicinity of atoms Pd2, Cl4, C48, and C54; the final map had no other significant features. A final analysis of variance between observed and calculated structure factors showed some dependence on amplitude and little dependence on resolution.

### Fractional atomic coordinates and isotropic or equivalent isotropic displacement parameters (Å^2^)

**Table:**

	*x*	*y*	*z*	*U*_iso_*/*U*_eq_	Occ. (<1)
Pd1	−0.003487 (12)	0.267501 (13)	0.482122 (12)	0.02435 (6)	
P1	0.08444 (4)	0.21236 (4)	0.56213 (4)	0.02565 (16)	
P2	−0.04231 (4)	0.28693 (4)	0.60477 (4)	0.02460 (16)	
Cl1	0.04979 (5)	0.24648 (5)	0.35578 (4)	0.03729 (18)	
Cl2	−0.11120 (4)	0.32869 (5)	0.42864 (5)	0.04003 (19)	
C1	0.1820 (3)	0.2426 (6)	0.5586 (6)	0.0332 (16)	0.58 (2)
C2	0.2345 (4)	0.2155 (5)	0.6137 (5)	0.0372 (16)	0.58 (2)
C3	0.3084 (4)	0.2406 (6)	0.6049 (7)	0.048 (2)	0.58 (2)
H3	0.3456	0.2234	0.6416	0.058*	0.58 (2)
C4	0.3277 (5)	0.2894 (8)	0.5444 (7)	0.053 (2)	0.58 (2)
H4	0.3786	0.3046	0.5392	0.063*	0.58 (2)
C5	0.2770 (6)	0.3175 (7)	0.4908 (6)	0.052 (2)	0.58 (2)
H5	0.2918	0.3526	0.4498	0.063*	0.58 (2)
C6	0.2030 (5)	0.2932 (7)	0.4977 (7)	0.0425 (19)	0.58 (2)
H6	0.1666	0.3113	0.4607	0.051*	0.58 (2)
C7	0.2183 (4)	0.1629 (5)	0.6826 (5)	0.0378 (18)	0.58 (2)
H7A	0.1811	0.1886	0.7164	0.057*	0.58 (2)
H7B	0.2644	0.1538	0.7129	0.057*	0.58 (2)
H7C	0.1987	0.1117	0.6637	0.057*	0.58 (2)
C1B	0.1795 (4)	0.2485 (8)	0.5497 (8)	0.033 (2)	0.42 (2)
C2B	0.2402 (6)	0.2222 (7)	0.5947 (8)	0.039 (2)	0.42 (2)
C3B	0.3100 (6)	0.2564 (7)	0.5810 (9)	0.045 (2)	0.42 (2)
H3B	0.3523	0.2395	0.6110	0.054*	0.42 (2)
C4B	0.3181 (7)	0.3139 (9)	0.5247 (9)	0.048 (2)	0.42 (2)
H4B	0.3663	0.3359	0.5158	0.057*	0.42 (2)
C5B	0.2591 (7)	0.3407 (8)	0.4811 (8)	0.042 (2)	0.42 (2)
H5B	0.2658	0.3815	0.4429	0.050*	0.42 (2)
C6B	0.1889 (7)	0.3078 (9)	0.4931 (9)	0.036 (2)	0.42 (2)
H6B	0.1473	0.3258	0.4627	0.043*	0.42 (2)
C7B	0.2347 (6)	0.1602 (8)	0.6572 (9)	0.049 (2)	0.42 (2)
H7D	0.2828	0.1556	0.6850	0.074*	0.42 (2)
H7E	0.2219	0.1088	0.6331	0.074*	0.42 (2)
H7F	0.1958	0.1753	0.6948	0.074*	0.42 (2)
C8	0.07714 (16)	0.10450 (18)	0.57255 (18)	0.0312 (7)	
C9	0.10714 (17)	0.05362 (19)	0.51544 (19)	0.0355 (7)	
C10	0.0951 (2)	−0.0281 (2)	0.5250 (2)	0.0457 (9)	
H10	0.1140	−0.0638	0.4864	0.055*	
C11	0.0573 (2)	−0.0584 (2)	0.5875 (3)	0.0521 (10)	
H11	0.0499	−0.1144	0.5916	0.063*	
C12	0.0297 (2)	−0.0090 (2)	0.6445 (2)	0.0502 (9)	
H12	0.0042	−0.0302	0.6887	0.060*	
C13	0.03945 (19)	0.07127 (18)	0.6368 (2)	0.0405 (8)	
H13	0.0200	0.1057	0.6762	0.049*	
C14	0.1503 (2)	0.0829 (2)	0.4460 (2)	0.0465 (9)	
H14A	0.1231	0.1269	0.4208	0.070*	
H14B	0.1565	0.0393	0.4081	0.070*	
H14C	0.1996	0.1016	0.4637	0.070*	
C15	0.04670 (16)	0.26069 (16)	0.65182 (16)	0.0273 (6)	
H15A	0.0409	0.2231	0.6965	0.033*	
H15B	0.0760	0.3080	0.6686	0.033*	
C16	−0.11518 (16)	0.22124 (17)	0.63808 (17)	0.0270 (6)	
C17	−0.17584 (18)	0.2076 (2)	0.58852 (19)	0.0377 (7)	
H17	−0.1784	0.2327	0.5383	0.045*	
C18	−0.2327 (2)	0.1573 (2)	0.6124 (2)	0.0505 (10)	
H18	−0.2753	0.1496	0.5794	0.061*	
C19	−0.2277 (2)	0.1185 (2)	0.6839 (2)	0.0477 (9)	
H19	−0.2660	0.0826	0.6992	0.057*	
C20	−0.1676 (2)	0.1314 (2)	0.7332 (2)	0.0430 (8)	
H20	−0.1646	0.1042	0.7823	0.052*	
C21	−0.11141 (18)	0.1838 (2)	0.71164 (18)	0.0356 (7)	
H21	−0.0707	0.1942	0.7466	0.043*	
C22	−0.06719 (17)	0.38759 (18)	0.63035 (17)	0.0294 (6)	
C23	−0.0217 (2)	0.4501 (2)	0.6063 (2)	0.0433 (8)	
H23	0.0230	0.4393	0.5783	0.052*	
C24	−0.0414 (2)	0.5281 (2)	0.6231 (2)	0.0510 (9)	
H24	−0.0095	0.5706	0.6077	0.061*	
C25	−0.1066 (2)	0.5444 (2)	0.6616 (2)	0.0505 (9)	
H25	−0.1205	0.5982	0.6717	0.061*	
C26	−0.1520 (2)	0.4832 (2)	0.6855 (3)	0.0575 (11)	
H26	−0.1971	0.4949	0.7125	0.069*	
C27	−0.1326 (2)	0.4046 (2)	0.6708 (2)	0.0460 (9)	
H27	−0.1639	0.3625	0.6884	0.055*	
Pd2	0.488381 (12)	0.480723 (13)	0.266672 (13)	0.02681 (7)	
P3	0.55839 (4)	0.58234 (4)	0.21988 (4)	0.02569 (16)	
P4	0.42890 (4)	0.59234 (5)	0.29718 (4)	0.02862 (17)	
Cl3	0.56527 (5)	0.37017 (5)	0.23698 (5)	0.0431 (2)	
Cl4	0.39319 (5)	0.39998 (6)	0.31809 (6)	0.0586 (3)	
C28	0.54532 (16)	0.60223 (19)	0.11524 (18)	0.0347 (7)	
C29	0.58273 (19)	0.5576 (2)	0.0591 (2)	0.0418 (8)	
C30	0.5684 (2)	0.5757 (2)	−0.0217 (2)	0.0530 (10)	
H30	0.5937	0.5467	−0.0616	0.064*	
C31	0.5194 (3)	0.6337 (3)	−0.0426 (2)	0.0617 (12)	
H31	0.5105	0.6440	−0.0970	0.074*	
C32	0.4827 (2)	0.6775 (3)	0.0124 (2)	0.0603 (11)	
H32	0.4485	0.7181	−0.0031	0.072*	
C33	0.4957 (2)	0.6623 (2)	0.08936 (19)	0.0497 (9)	
H33	0.4703	0.6934	0.1277	0.060*	
C34	0.6361 (2)	0.4946 (2)	0.0783 (2)	0.0529 (10)	
H34A	0.6162	0.4615	0.1209	0.079*	
H34B	0.6835	0.5186	0.0955	0.079*	
H34C	0.6445	0.4615	0.0316	0.079*	
C35	0.65584 (16)	0.58542 (18)	0.25143 (18)	0.0307 (7)	
C36	0.70975 (19)	0.6366 (2)	0.2212 (2)	0.0391 (8)	
C37	0.78383 (19)	0.6289 (2)	0.2510 (2)	0.0480 (9)	
H37	0.8226	0.6612	0.2301	0.058*	
C38	0.7998 (2)	0.5756 (2)	0.3093 (2)	0.0524 (10)	
H38	0.8499	0.5714	0.3279	0.063*	
C39	0.7466 (2)	0.5281 (2)	0.3418 (2)	0.0503 (9)	
H39	0.7588	0.4929	0.3840	0.060*	
C40	0.67476 (18)	0.5321 (2)	0.3125 (2)	0.0386 (7)	
H40	0.6373	0.4983	0.3338	0.046*	
C41	0.6961 (2)	0.6991 (3)	0.1625 (3)	0.0609 (11)	
H41A	0.6526	0.7309	0.1781	0.091*	
H41B	0.6863	0.6745	0.1109	0.091*	
H41C	0.7401	0.7337	0.1591	0.091*	
C42	0.50777 (17)	0.65964 (18)	0.27665 (17)	0.0318 (7)	
H42A	0.5349	0.6768	0.3249	0.038*	
H42B	0.4933	0.7065	0.2443	0.038*	
C43	0.4046 (2)	0.60883 (11)	0.3993 (2)	0.0342 (9)	0.838 (9)
C44	0.3333 (3)	0.6318 (3)	0.4212 (2)	0.0477 (12)	0.838 (9)
H44	0.2956	0.6395	0.3821	0.057*	0.838 (9)
C45	0.3168 (3)	0.6437 (3)	0.5005 (3)	0.0557 (15)	0.838 (9)
H45	0.2678	0.6588	0.5158	0.067*	0.838 (9)
C46	0.3716 (3)	0.6337 (2)	0.5561 (2)	0.0484 (13)	0.838 (9)
H46	0.3606	0.6430	0.6101	0.058*	0.838 (9)
C47	0.4423 (3)	0.6103 (3)	0.5352 (2)	0.0474 (12)	0.838 (9)
H47	0.4797	0.6032	0.5748	0.057*	0.838 (9)
C48	0.4594 (3)	0.5970 (3)	0.4567 (3)	0.0421 (10)	0.838 (9)
H48	0.5081	0.5801	0.4423	0.050*	0.838 (9)
C43B	0.3923 (12)	0.6025 (5)	0.3961 (7)	0.041 (3)	0.162 (9)
C44B	0.3159 (11)	0.5970 (13)	0.4095 (9)	0.043 (3)	0.162 (9)
H44B	0.2826	0.5878	0.3665	0.052*	0.162 (9)
C45B	0.2882 (11)	0.6049 (15)	0.4854 (10)	0.050 (3)	0.162 (9)
H45B	0.2359	0.6010	0.4944	0.060*	0.162 (9)
C46B	0.3361 (14)	0.6182 (11)	0.5473 (10)	0.050 (3)	0.162 (9)
H46B	0.3169	0.6237	0.5991	0.060*	0.162 (9)
C47B	0.4113 (14)	0.6237 (13)	0.5349 (10)	0.047 (3)	0.162 (9)
H47B	0.4441	0.6330	0.5783	0.057*	0.162 (9)
C48B	0.4406 (12)	0.6158 (12)	0.4593 (11)	0.044 (3)	0.162 (9)
H48B	0.4930	0.6195	0.4512	0.052*	0.162 (9)
C49	0.35162 (16)	0.61621 (19)	0.23292 (17)	0.0313 (7)	
C50	0.32022 (19)	0.5565 (2)	0.1869 (2)	0.0418 (8)	
H50	0.3380	0.5032	0.1912	0.050*	
C51	0.2627 (2)	0.5748 (2)	0.1346 (2)	0.0537 (10)	
H51	0.2412	0.5343	0.1024	0.064*	
C52	0.2368 (2)	0.6517 (3)	0.1295 (2)	0.0527 (10)	
H52	0.1971	0.6638	0.0939	0.063*	
C53	0.2674 (2)	0.7115 (2)	0.1749 (2)	0.0465 (9)	
H53	0.2489	0.7645	0.1705	0.056*	
C54	0.32488 (19)	0.6945 (2)	0.2270 (2)	0.0424 (8)	
H54	0.3462	0.7356	0.2585	0.051*	

### Atomic displacement parameters (Å^2^)

**Table:**

	*U*^11^	*U*^22^	*U*^33^	*U*^12^	*U*^13^	*U*^23^
Pd1	0.02804 (12)	0.02685 (12)	0.01816 (11)	−0.00370 (9)	0.00142 (8)	−0.00013 (8)
P1	0.0263 (4)	0.0273 (4)	0.0233 (4)	−0.0031 (3)	0.0014 (3)	−0.0008 (3)
P2	0.0238 (4)	0.0290 (4)	0.0211 (3)	−0.0058 (3)	0.0023 (3)	−0.0014 (3)
Cl1	0.0511 (5)	0.0393 (4)	0.0215 (3)	0.0050 (4)	0.0079 (3)	0.0004 (3)
Cl2	0.0319 (4)	0.0551 (5)	0.0330 (4)	−0.0009 (4)	−0.0036 (3)	0.0092 (4)
C1	0.030 (3)	0.033 (3)	0.037 (3)	−0.004 (3)	0.009 (3)	−0.009 (3)
C2	0.030 (3)	0.043 (3)	0.039 (4)	0.000 (2)	0.003 (2)	−0.013 (3)
C3	0.034 (3)	0.060 (4)	0.049 (5)	−0.002 (3)	0.005 (3)	−0.011 (3)
C4	0.036 (3)	0.065 (5)	0.058 (5)	−0.008 (3)	0.007 (3)	−0.009 (4)
C5	0.044 (4)	0.058 (5)	0.055 (4)	−0.009 (4)	0.013 (3)	−0.001 (4)
C6	0.038 (3)	0.049 (4)	0.041 (3)	−0.007 (3)	0.010 (3)	−0.007 (3)
C7	0.029 (3)	0.047 (3)	0.037 (4)	0.005 (3)	−0.002 (3)	−0.003 (3)
C1B	0.028 (3)	0.038 (4)	0.032 (4)	−0.005 (3)	0.006 (3)	−0.012 (3)
C2B	0.033 (3)	0.046 (4)	0.039 (4)	−0.002 (3)	0.007 (3)	−0.014 (3)
C3B	0.033 (3)	0.057 (4)	0.045 (5)	−0.006 (3)	0.005 (4)	−0.010 (4)
C4B	0.034 (4)	0.057 (5)	0.052 (5)	−0.011 (4)	0.011 (4)	−0.008 (4)
C5B	0.037 (4)	0.042 (5)	0.046 (4)	−0.010 (4)	0.016 (4)	−0.009 (4)
C6B	0.032 (4)	0.039 (4)	0.037 (4)	−0.005 (3)	0.011 (3)	−0.010 (3)
C7B	0.040 (4)	0.059 (4)	0.049 (5)	0.003 (4)	−0.001 (4)	−0.010 (4)
C8	0.0303 (15)	0.0284 (16)	0.0348 (16)	−0.0030 (13)	−0.0090 (13)	0.0020 (13)
C9	0.0317 (16)	0.0336 (17)	0.0411 (18)	0.0027 (13)	−0.0133 (14)	−0.0029 (14)
C10	0.045 (2)	0.0321 (18)	0.060 (2)	0.0054 (15)	−0.0196 (18)	−0.0081 (17)
C11	0.048 (2)	0.0297 (18)	0.079 (3)	−0.0077 (16)	−0.023 (2)	0.0061 (19)
C12	0.051 (2)	0.042 (2)	0.057 (2)	−0.0136 (17)	−0.0108 (18)	0.0180 (18)
C13	0.0424 (19)	0.0363 (18)	0.0427 (19)	−0.0055 (15)	−0.0046 (15)	0.0062 (15)
C14	0.054 (2)	0.040 (2)	0.046 (2)	0.0088 (17)	0.0043 (17)	−0.0100 (16)
C15	0.0266 (14)	0.0339 (16)	0.0212 (14)	−0.0057 (12)	0.0004 (11)	−0.0032 (12)
C16	0.0267 (15)	0.0286 (15)	0.0257 (14)	−0.0070 (12)	0.0053 (11)	−0.0037 (12)
C17	0.0372 (18)	0.0421 (19)	0.0336 (17)	−0.0103 (15)	−0.0037 (14)	0.0057 (14)
C18	0.041 (2)	0.062 (2)	0.049 (2)	−0.0247 (18)	−0.0103 (16)	0.0066 (19)
C19	0.044 (2)	0.053 (2)	0.046 (2)	−0.0241 (17)	0.0058 (16)	0.0020 (17)
C20	0.049 (2)	0.048 (2)	0.0314 (17)	−0.0174 (17)	0.0065 (15)	0.0052 (15)
C21	0.0348 (17)	0.0429 (19)	0.0293 (16)	−0.0090 (14)	0.0029 (13)	0.0005 (14)
C22	0.0319 (16)	0.0300 (16)	0.0265 (15)	−0.0049 (13)	0.0015 (12)	−0.0014 (12)
C23	0.0406 (19)	0.0427 (19)	0.047 (2)	−0.0079 (16)	0.0139 (16)	0.0009 (16)
C24	0.063 (2)	0.0328 (19)	0.058 (2)	−0.0119 (17)	0.0082 (19)	0.0061 (17)
C25	0.067 (3)	0.0334 (19)	0.051 (2)	0.0003 (18)	0.0089 (19)	−0.0042 (16)
C26	0.058 (2)	0.044 (2)	0.071 (3)	0.0016 (18)	0.028 (2)	−0.013 (2)
C27	0.046 (2)	0.0380 (19)	0.054 (2)	−0.0086 (16)	0.0223 (17)	−0.0094 (16)
Pd2	0.02488 (12)	0.02596 (12)	0.02958 (12)	−0.00231 (9)	0.00016 (9)	0.00152 (9)
P3	0.0237 (4)	0.0266 (4)	0.0268 (4)	0.0003 (3)	0.0015 (3)	0.0004 (3)
P4	0.0222 (4)	0.0352 (4)	0.0285 (4)	0.0032 (3)	0.0007 (3)	−0.0015 (3)
Cl3	0.0546 (5)	0.0288 (4)	0.0457 (5)	0.0108 (4)	−0.0038 (4)	−0.0027 (3)
Cl4	0.0459 (5)	0.0628 (6)	0.0670 (6)	−0.0236 (5)	−0.0002 (4)	0.0214 (5)
C28	0.0338 (17)	0.0390 (18)	0.0313 (16)	−0.0104 (14)	−0.0007 (13)	0.0032 (14)
C29	0.0385 (18)	0.043 (2)	0.0439 (19)	−0.0111 (15)	0.0035 (15)	−0.0048 (16)
C30	0.063 (3)	0.059 (2)	0.038 (2)	−0.021 (2)	0.0048 (18)	−0.0074 (18)
C31	0.073 (3)	0.070 (3)	0.042 (2)	−0.020 (2)	−0.011 (2)	0.010 (2)
C32	0.061 (3)	0.063 (3)	0.056 (3)	−0.004 (2)	−0.015 (2)	0.017 (2)
C33	0.048 (2)	0.053 (2)	0.048 (2)	0.0004 (18)	−0.0037 (17)	0.0134 (18)
C34	0.060 (2)	0.054 (2)	0.045 (2)	−0.0025 (19)	0.0065 (18)	−0.0048 (18)
C35	0.0266 (15)	0.0328 (16)	0.0326 (16)	0.0000 (12)	0.0046 (12)	−0.0094 (13)
C36	0.0393 (18)	0.0353 (18)	0.0427 (19)	−0.0064 (14)	0.0080 (15)	−0.0084 (15)
C37	0.0345 (18)	0.049 (2)	0.061 (2)	−0.0116 (16)	0.0045 (17)	−0.0186 (19)
C38	0.040 (2)	0.049 (2)	0.068 (3)	0.0023 (17)	−0.0109 (18)	−0.019 (2)
C39	0.050 (2)	0.048 (2)	0.053 (2)	0.0071 (18)	−0.0131 (18)	−0.0089 (18)
C40	0.0342 (17)	0.0411 (19)	0.0406 (18)	0.0063 (14)	−0.0039 (14)	−0.0046 (15)
C41	0.056 (2)	0.060 (3)	0.067 (3)	−0.016 (2)	−0.001 (2)	0.002 (2)
C42	0.0333 (16)	0.0255 (15)	0.0367 (17)	0.0019 (13)	0.0037 (13)	−0.0019 (13)
C43	0.031 (2)	0.042 (2)	0.0299 (18)	0.0071 (16)	0.0020 (15)	0.0001 (16)
C44	0.035 (2)	0.072 (3)	0.036 (2)	0.012 (2)	−0.0003 (17)	0.002 (2)
C45	0.047 (3)	0.079 (3)	0.042 (2)	0.023 (3)	0.010 (2)	0.002 (2)
C46	0.057 (3)	0.057 (3)	0.032 (2)	0.014 (2)	0.005 (2)	0.0037 (19)
C47	0.051 (3)	0.055 (3)	0.035 (2)	0.011 (2)	−0.010 (2)	0.0057 (19)
C48	0.040 (2)	0.049 (2)	0.0375 (19)	0.0142 (19)	−0.0023 (17)	0.0002 (19)
C43B	0.036 (5)	0.054 (5)	0.032 (4)	0.015 (5)	0.000 (4)	0.002 (5)
C44B	0.036 (5)	0.060 (6)	0.033 (5)	0.014 (5)	0.007 (4)	0.001 (5)
C45B	0.045 (5)	0.069 (6)	0.036 (5)	0.016 (5)	0.007 (4)	0.005 (5)
C46B	0.050 (5)	0.065 (6)	0.036 (5)	0.020 (5)	0.006 (5)	0.002 (5)
C47B	0.049 (5)	0.057 (5)	0.035 (4)	0.015 (5)	−0.001 (5)	0.002 (5)
C48B	0.041 (5)	0.053 (5)	0.036 (4)	0.014 (5)	0.000 (4)	0.000 (5)
C49	0.0237 (14)	0.0420 (18)	0.0282 (15)	0.0010 (13)	0.0032 (12)	0.0024 (13)
C50	0.0366 (18)	0.044 (2)	0.0443 (19)	−0.0012 (15)	−0.0049 (15)	0.0058 (16)
C51	0.051 (2)	0.057 (2)	0.053 (2)	−0.0087 (19)	−0.0182 (18)	−0.0015 (19)
C52	0.038 (2)	0.066 (3)	0.054 (2)	0.0007 (18)	−0.0110 (17)	0.022 (2)
C53	0.0390 (19)	0.048 (2)	0.053 (2)	0.0085 (16)	0.0016 (16)	0.0133 (18)
C54	0.0365 (18)	0.043 (2)	0.048 (2)	0.0033 (15)	−0.0014 (15)	0.0033 (16)

### Geometric parameters (Å, °)

**Table:**

Pd1—P2	2.2197 (7)	C27—H27	0.9500
Pd1—P1	2.2646 (8)	Pd2—P4	2.2146 (8)
Pd1—Cl2	2.3564 (8)	Pd2—P3	2.2604 (8)
Pd1—Cl1	2.3758 (7)	Pd2—Cl4	2.3469 (9)
P1—C1B	1.817 (6)	Pd2—Cl3	2.3632 (8)
P1—C1	1.818 (5)	P3—C28	1.817 (3)
P1—C8	1.821 (3)	P3—C35	1.819 (3)
P1—C15	1.853 (3)	P3—C42	1.853 (3)
P1—P2	2.6912 (11)	P3—P4	2.6738 (10)
P2—C22	1.798 (3)	P4—C49	1.797 (3)
P2—C16	1.800 (3)	P4—C43	1.808 (4)
P2—C15	1.828 (3)	P4—C43B	1.812 (9)
C1—C6	1.389 (7)	P4—C42	1.841 (3)
C1—C2	1.394 (7)	C28—C29	1.386 (5)
C2—C3	1.395 (7)	C28—C33	1.410 (5)
C2—C7	1.493 (7)	C29—C30	1.423 (5)
C3—C4	1.359 (9)	C29—C34	1.457 (5)
C3—H3	0.9500	C30—C31	1.353 (6)
C4—C5	1.360 (9)	C30—H30	0.9500
C4—H4	0.9500	C31—C32	1.360 (6)
C5—C6	1.390 (7)	C31—H31	0.9500
C5—H5	0.9500	C32—C33	1.346 (5)
C6—H6	0.9500	C32—H32	0.9500
C7—H7A	0.9800	C33—H33	0.9500
C7—H7B	0.9800	C34—H34A	0.9800
C7—H7C	0.9800	C34—H34B	0.9800
C1B—C6B	1.392 (8)	C34—H34C	0.9800
C1B—C2B	1.394 (8)	C35—C36	1.391 (4)
C2B—C3B	1.395 (8)	C35—C40	1.406 (5)
C2B—C7B	1.488 (9)	C36—C37	1.420 (5)
C3B—C4B	1.364 (10)	C36—C41	1.463 (5)
C3B—H3B	0.9500	C37—C38	1.359 (6)
C4B—C5B	1.360 (10)	C37—H37	0.9500
C4B—H4B	0.9500	C38—C39	1.360 (6)
C5B—C6B	1.386 (8)	C38—H38	0.9500
C5B—H5B	0.9500	C39—C40	1.375 (5)
C6B—H6B	0.9500	C39—H39	0.9500
C7B—H7D	0.9800	C40—H40	0.9500
C7B—H7E	0.9800	C41—H41A	0.9800
C7B—H7F	0.9800	C41—H41B	0.9800
C8—C13	1.399 (4)	C41—H41C	0.9800
C8—C9	1.400 (4)	C42—H42A	0.9900
C9—C10	1.396 (5)	C42—H42B	0.9900
C9—C14	1.494 (5)	C43—C44	1.385 (5)
C10—C11	1.359 (6)	C43—C48	1.390 (5)
C10—H10	0.9500	C44—C45	1.392 (5)
C11—C12	1.366 (6)	C44—H44	0.9500
C11—H11	0.9500	C45—C46	1.364 (5)
C12—C13	1.362 (5)	C45—H45	0.9500
C12—H12	0.9500	C46—C47	1.373 (5)
C13—H13	0.9500	C46—H46	0.9500
C14—H14A	0.9800	C47—C48	1.385 (5)
C14—H14B	0.9800	C47—H47	0.9500
C14—H14C	0.9800	C48—H48	0.9500
C15—H15A	0.9900	C43B—C44B	1.388 (8)
C15—H15B	0.9900	C43B—C48B	1.388 (8)
C16—C17	1.385 (4)	C44B—C45B	1.389 (9)
C16—C21	1.396 (4)	C44B—H44B	0.9500
C17—C18	1.383 (5)	C45B—C46B	1.366 (9)
C17—H17	0.9500	C45B—H45B	0.9500
C18—C19	1.378 (5)	C46B—C47B	1.366 (9)
C18—H18	0.9500	C46B—H46B	0.9500
C19—C20	1.371 (5)	C47B—C48B	1.392 (9)
C19—H19	0.9500	C47B—H47B	0.9500
C20—C21	1.386 (4)	C48B—H48B	0.9500
C20—H20	0.9500	C49—C50	1.384 (5)
C21—H21	0.9500	C49—C54	1.400 (5)
C22—C23	1.389 (4)	C50—C51	1.387 (5)
C22—C27	1.390 (4)	C50—H50	0.9500
C23—C24	1.383 (5)	C51—C52	1.370 (6)
C23—H23	0.9500	C51—H51	0.9500
C24—C25	1.367 (5)	C52—C53	1.375 (5)
C24—H24	0.9500	C52—H52	0.9500
C25—C26	1.371 (5)	C53—C54	1.379 (5)
C25—H25	0.9500	C53—H53	0.9500
C26—C27	1.385 (5)	C54—H54	0.9500
C26—H26	0.9500		
			
P2—Pd1—P1	73.75 (3)	P4—Pd2—P3	73.37 (3)
P2—Pd1—Cl2	92.04 (3)	P4—Pd2—Cl4	92.83 (4)
P1—Pd1—Cl2	165.68 (3)	P3—Pd2—Cl4	165.80 (4)
P2—Pd1—Cl1	174.57 (3)	P4—Pd2—Cl3	173.13 (3)
P1—Pd1—Cl1	101.39 (3)	P3—Pd2—Cl3	101.03 (3)
Cl2—Pd1—Cl1	92.88 (3)	Cl4—Pd2—Cl3	92.96 (4)
C1B—P1—C8	114.2 (5)	C28—P3—C35	113.40 (14)
C1—P1—C8	110.4 (4)	C28—P3—C42	108.57 (15)
C1B—P1—C15	107.2 (5)	C35—P3—C42	107.34 (13)
C1—P1—C15	105.1 (4)	C28—P3—Pd2	114.39 (10)
C8—P1—C15	109.11 (14)	C35—P3—Pd2	116.74 (11)
C1B—P1—Pd1	116.1 (4)	C42—P3—Pd2	94.05 (10)
C1—P1—Pd1	121.9 (3)	C28—P3—P4	111.16 (10)
C8—P1—Pd1	114.40 (9)	C35—P3—P4	133.25 (10)
C15—P1—Pd1	93.27 (9)	Pd2—P3—P4	52.53 (2)
C1B—P1—P2	131.8 (5)	C49—P4—C43	110.86 (16)
C1—P1—P2	133.6 (3)	C49—P4—C43B	104.9 (7)
C8—P1—P2	111.94 (10)	C49—P4—C42	109.60 (14)
Pd1—P1—P2	52.36 (2)	C43—P4—C42	106.10 (13)
C22—P2—C16	108.49 (14)	C43B—P4—C42	113.6 (6)
C22—P2—C15	109.67 (13)	C49—P4—Pd2	114.51 (11)
C16—P2—C15	110.24 (14)	C43—P4—Pd2	118.15 (10)
C22—P2—Pd1	116.29 (10)	C43B—P4—Pd2	118.2 (3)
C16—P2—Pd1	115.86 (9)	C42—P4—Pd2	95.94 (10)
C15—P2—Pd1	95.48 (9)	C49—P4—P3	112.43 (10)
C22—P2—P1	135.28 (10)	C43—P4—P3	133.92 (13)
C16—P2—P1	114.41 (10)	C43B—P4—P3	141.2 (7)
Pd1—P2—P1	53.89 (2)	Pd2—P4—P3	54.10 (2)
C6—C1—C2	120.8 (5)	C29—C28—C33	118.6 (3)
C6—C1—P1	117.3 (6)	C29—C28—P3	120.7 (3)
C2—C1—P1	121.9 (6)	C33—C28—P3	120.7 (3)
C1—C2—C3	117.6 (6)	C28—C29—C30	117.4 (3)
C1—C2—C7	125.6 (6)	C28—C29—C34	123.7 (3)
C3—C2—C7	116.8 (6)	C30—C29—C34	118.8 (3)
C4—C3—C2	120.5 (7)	C31—C30—C29	121.1 (4)
C4—C3—H3	119.8	C31—C30—H30	119.4
C2—C3—H3	119.8	C29—C30—H30	119.4
C3—C4—C5	122.7 (6)	C30—C31—C32	121.5 (4)
C3—C4—H4	118.6	C30—C31—H31	119.2
C5—C4—H4	118.6	C32—C31—H31	119.2
C4—C5—C6	118.2 (7)	C33—C32—C31	118.8 (4)
C4—C5—H5	120.9	C33—C32—H32	120.6
C6—C5—H5	120.9	C31—C32—H32	120.6
C1—C6—C5	120.2 (7)	C32—C33—C28	122.6 (4)
C1—C6—H6	119.9	C32—C33—H33	118.7
C5—C6—H6	119.9	C28—C33—H33	118.7
C6B—C1B—C2B	120.3 (7)	C29—C34—H34A	109.5
C6B—C1B—P1	115.9 (8)	C29—C34—H34B	109.5
C2B—C1B—P1	123.8 (7)	H34A—C34—H34B	109.5
C1B—C2B—C3B	118.3 (8)	C29—C34—H34C	109.5
C1B—C2B—C7B	123.7 (8)	H34A—C34—H34C	109.5
C3B—C2B—C7B	118.0 (8)	H34B—C34—H34C	109.5
C4B—C3B—C2B	120.4 (8)	C36—C35—C40	120.0 (3)
C4B—C3B—H3B	119.8	C36—C35—P3	125.0 (3)
C2B—C3B—H3B	119.8	C40—C35—P3	115.0 (2)
C5B—C4B—C3B	121.8 (8)	C35—C36—C37	117.3 (3)
C5B—C4B—H4B	119.1	C35—C36—C41	125.4 (3)
C3B—C4B—H4B	119.1	C37—C36—C41	117.2 (3)
C4B—C5B—C6B	119.2 (8)	C38—C37—C36	120.5 (3)
C4B—C5B—H5B	120.4	C38—C37—H37	119.7
C6B—C5B—H5B	120.4	C36—C37—H37	119.7
C5B—C6B—C1B	120.0 (8)	C37—C38—C39	122.4 (4)
C5B—C6B—H6B	120.0	C37—C38—H38	118.8
C1B—C6B—H6B	120.0	C39—C38—H38	118.8
C2B—C7B—H7D	109.5	C38—C39—C40	118.6 (4)
C2B—C7B—H7E	109.5	C38—C39—H39	120.7
H7D—C7B—H7E	109.5	C40—C39—H39	120.7
C2B—C7B—H7F	109.5	C39—C40—C35	121.0 (3)
H7D—C7B—H7F	109.5	C39—C40—H40	119.5
H7E—C7B—H7F	109.5	C35—C40—H40	119.5
C13—C8—C9	119.0 (3)	C36—C41—H41A	109.5
C13—C8—P1	120.4 (2)	C36—C41—H41B	109.5
C9—C8—P1	120.6 (2)	H41A—C41—H41B	109.5
C10—C9—C8	117.2 (3)	C36—C41—H41C	109.5
C10—C9—C14	119.6 (3)	H41A—C41—H41C	109.5
C8—C9—C14	123.2 (3)	H41B—C41—H41C	109.5
C11—C10—C9	122.3 (4)	P4—C42—P3	92.73 (14)
C11—C10—H10	118.8	P4—C42—H42A	113.2
C9—C10—H10	118.8	P3—C42—H42A	113.2
C10—C11—C12	120.5 (3)	P4—C42—H42B	113.2
C10—C11—H11	119.7	P3—C42—H42B	113.2
C12—C11—H11	119.7	H42A—C42—H42B	110.5
C13—C12—C11	118.9 (4)	C44—C43—C48	119.8 (3)
C13—C12—H12	120.5	C44—C43—P4	121.9 (3)
C11—C12—H12	120.5	C48—C43—P4	118.3 (3)
C12—C13—C8	122.0 (3)	C43—C44—C45	120.1 (4)
C12—C13—H13	119.0	C43—C44—H44	120.0
C8—C13—H13	119.0	C45—C44—H44	120.0
C9—C14—H14A	109.5	C46—C45—C44	119.5 (4)
C9—C14—H14B	109.5	C46—C45—H45	120.2
H14A—C14—H14B	109.5	C44—C45—H45	120.2
C9—C14—H14C	109.5	C45—C46—C47	121.0 (4)
H14A—C14—H14C	109.5	C45—C46—H46	119.5
H14B—C14—H14C	109.5	C47—C46—H46	119.5
P2—C15—P1	93.95 (13)	C46—C47—C48	120.3 (4)
P2—C15—H15A	112.9	C46—C47—H47	119.9
P1—C15—H15A	112.9	C48—C47—H47	119.9
P2—C15—H15B	112.9	C47—C48—C43	119.3 (4)
P1—C15—H15B	112.9	C47—C48—H48	120.3
H15A—C15—H15B	110.4	C43—C48—H48	120.3
C17—C16—C21	119.9 (3)	C44B—C43B—C48B	119.4 (10)
C17—C16—P2	118.4 (2)	C44B—C43B—P4	120.5 (12)
C21—C16—P2	121.6 (2)	C48B—C43B—P4	120.1 (13)
C18—C17—C16	119.8 (3)	C43B—C44B—C45B	120.3 (11)
C18—C17—H17	120.1	C43B—C44B—H44B	119.9
C16—C17—H17	120.1	C45B—C44B—H44B	119.9
C19—C18—C17	120.1 (3)	C46B—C45B—C44B	120.0 (11)
C19—C18—H18	120.0	C46B—C45B—H45B	120.0
C17—C18—H18	120.0	C44B—C45B—H45B	120.0
C20—C19—C18	120.5 (3)	C45B—C46B—C47B	120.4 (12)
C20—C19—H19	119.8	C45B—C46B—H46B	119.8
C18—C19—H19	119.8	C47B—C46B—H46B	119.8
C19—C20—C21	120.3 (3)	C46B—C47B—C48B	120.8 (12)
C19—C20—H20	119.8	C46B—C47B—H47B	119.6
C21—C20—H20	119.8	C48B—C47B—H47B	119.6
C20—C21—C16	119.3 (3)	C43B—C48B—C47B	119.3 (11)
C20—C21—H21	120.3	C43B—C48B—H48B	120.4
C16—C21—H21	120.3	C47B—C48B—H48B	120.4
C23—C22—C27	119.1 (3)	C50—C49—C54	120.0 (3)
C23—C22—P2	119.4 (2)	C50—C49—P4	119.1 (3)
C27—C22—P2	121.5 (2)	C54—C49—P4	120.8 (3)
C24—C23—C22	120.1 (3)	C49—C50—C51	119.6 (3)
C24—C23—H23	119.9	C49—C50—H50	120.2
C22—C23—H23	119.9	C51—C50—H50	120.2
C25—C24—C23	120.4 (3)	C52—C51—C50	119.8 (4)
C25—C24—H24	119.8	C52—C51—H51	120.1
C23—C24—H24	119.8	C50—C51—H51	120.1
C24—C25—C26	120.0 (3)	C51—C52—C53	121.2 (3)
C24—C25—H25	120.0	C51—C52—H52	119.4
C26—C25—H25	120.0	C53—C52—H52	119.4
C25—C26—C27	120.6 (4)	C52—C53—C54	119.9 (3)
C25—C26—H26	119.7	C52—C53—H53	120.1
C27—C26—H26	119.7	C54—C53—H53	120.1
C26—C27—C22	119.7 (3)	C53—C54—C49	119.5 (3)
C26—C27—H27	120.1	C53—C54—H54	120.3
C22—C27—H27	120.1	C49—C54—H54	120.3

### Hydrogen-bond geometry (Å, °)

**Table:**

*D*—H···*A*	*D*—H	H···*A*	*D*···*A*	*D*—H···*A*
C14—H14A···Cl1	0.98	2.63	3.612 (4)	179
C34—H34A···Cl3	0.98	2.66	3.637 (4)	179
